# Differential sensitivity to warming and hypoxia during development and long-term effects of developmental exposure in early life stage Chinook salmon

**DOI:** 10.1093/conphys/coab054

**Published:** 2021-07-08

**Authors:** Annelise M Del Rio, Gabriella N Mukai, Benjamin T Martin, Rachel C Johnson, Nann A Fangue, Joshua A Israel, Anne E Todgham

**Affiliations:** 1 Department of Animal Science, University of California Davis, Davis, CA 95616, USA; 2 Department of Biology, University of Hawai’i at Mānoa, Honolulu, HI 96822, USA; 3 University of California Santa Cruz, Cooperative Institute for Marine Ecosystems and Climate (CIMEC), Santa Cruz, CA 95064, USA; 4 Southwest Fisheries Science Center, National Marine Fisheries Service, National Oceanic and Atmospheric Administration, 110 Shaffer Road, Santa Cruz, CA 95060, USA; 5 Institute for Biodiversity and Ecosystem Dynamics, University of Amsterdam, 1098 XH Amsterdam, the Netherlands; 6 Department of Wildlife, Fish, and Conservation Biology, University of California Davis, Davis, CA 95616, USA; 7 Bay-Delta Office, U.S. Bureau of Reclamation, Sacramento, CA 95825, USA

**Keywords:** Carry-over effects, developmental windows, hypoxia, multiple stressors

## Abstract

Warming and hypoxia are two stressors commonly found within natural salmon redds that are likely to co-occur. Warming and hypoxia can interact physiologically, but their combined effects during fish development remain poorly studied, particularly stage-specific effects and potential carry-over effects. To test the impacts of warm water temperature and hypoxia as individual and combined developmental stressors, late fall-run Chinook salmon embryos were reared in 10 treatments from fertilization through hatching with two temperatures [10°C (ambient) and 14°C (warm)], two dissolved oxygen saturation levels [normoxia (100% air saturation, 10.4–11.4 mg O_2_/l) and hypoxia (50% saturation, 5.5 mg O_2_/l)] and three exposure times (early [eyed stage], late [silver-eyed stage] and chronic [fertilization through hatching]). After hatching, all treatments were transferred to control conditions (10°C and 100% air saturation) through the fry stage. To study stage-specific effects of stressor exposure we measured routine metabolic rate (RMR) at two embryonic stages, hatching success and growth. To evaluate carry-over effects, where conditions during one life stage influence performance in a later stage, RMR of all treatments was measured in control conditions at two post-hatch stages and acute stress tolerance was measured at the fry stage. We found evidence of stage-specific effects of both stressors during exposure and carry-over effects on physiological performance. Both individual stressors affected RMR, growth and developmental rate while multiple stressors late in development reduced hatching success. RMR post-hatch showed persistent effects of embryonic stressor exposure that may underlie differences observed in developmental timing and acute stress tolerance. The responses to stressors that varied by stage during development suggest that stage-specific management efforts could support salmon embryo survival. The persistent carry-over effects also indicate that considering sub-lethal effects of developmental stressor exposure may be important to understanding how climate change influences the performance of salmon across life stages.

## Teaser

We reared Chinook salmon embryos under 10 treatments that varied by stressor (warm temperature, hypoxia or both) and the timing of stress exposure (early, late or chronically during embryonic development) and found stage-specific sensitivity. We also investigated carry-over effects of developmental stressors and the management implications of short- and long-term sensitivity.

## Introduction

The vulnerability of a species to climate warming is largely determined by the most thermally sensitive life stage, as thermal tolerance changes throughout ontogeny for many fish species. Early life stages of fish are often the most sensitive to climate change stressors due to their limited capacity for behavioural responses, less developed stress tolerance mechanisms and tighter metabolic constraints (Pankhurst and Munday, 2011.; Rombough, 2011). In addition to warming, instances of hypoxia [low dissolved oxygen (DO)] in aquatic and marine environments are increasing in frequency and severity globally due to climate change (Breitburg *et al.*, 2018). Since stressors such as warming and hypoxia naturally co-occur in the habitats of fish embryos and larvae, studies that employ a multiple stressor experimental framework will better predict how fish populations will respond to climate change (Todgham and Stillman, 2013). As the survival and performance of early life stages may have large impacts on recruitment and population size in fishes, there is a growing need to study the effects of multiple climate change stressors within and across developmental stages.

Warming and hypoxia frequently co-occur because warm water holds less oxygen at saturation. Warming and hypoxia also interact on a physiological level in fishes because of competing metabolic effects (Fry, 1971). As a controlling factor, temperature increases standard and maximum metabolic rate, and in turn aerobic scope, until an upper limit is reached (McBryan *et al.*, 2013). In contrast, hypoxia is a limiting factor that reduces the amount of oxygen available to support the increased metabolic demand caused by warming. Hypoxia first limits maximum metabolic rate and consequently reduces aerobic scope. Early life stages of fishes already have lower aerobic scopes compared to juveniles and adults, making it more challenging to cope with further restrictions imposed by environmental stressors (Killen *et al.*, 2007; Dahlke *et al.*, 2020). Fish commonly respond to changes in temperature or DO by adjusting their oxygen uptake and consumption to balance demand, but early life stages are limited in their ability to adjust metabolically and have underdeveloped cardiorespiratory systems (Rombough, 2011). Fish embryos have finite energy reserves in the form of yolk, so changes to aerobic scope by warming and hypoxia can alter the amount of energy available for crucial processes like growth and development (Sokolova, 2013).

For fishes, the response to stressors such as warming and hypoxia can be stage specific (Mueller *et al.*, 2015; Bloomer *et al.*, 2016; Wood *et al.*, 2019). The response to stressors during development is largely dependent on the interaction between the duration of the stressor and the developmental timing of stress exposure (Eyck *et al.*, 2019). Later embryonic stages are often more sensitive to warming and hypoxia, potentially due to the increasing oxygen demand as development and tissue growth progresses with time as well as limitations of oxygen diffusion across the egg membrane (Rombough, 1988b; Martin *et al.,* 2020). However, very few studies have investigated stage-dependent sensitivity during exposure to multiple stressors before and after hatching in fishes.

Many ectotherms acclimate to environmental changes through mechanisms of physiological plasticity—the ability to modify physiological processes to maintain performance (Seebacher *et al.*, 2015). Early life stages of fishes undergo substantial changes in physiology and morphology during development, which can allow for organisms to better tailor their physiology to their environment. Acclimation through physiological and developmental plasticity can result in changes to survival, growth, swimming performance, behaviour, physiology or fitness (Vagner *et al.*, 2019). While acclimation resulting from physiological plasticity is reversible and repeatable, developmental plasticity is specific to early life stages and has persistent phenotypic effects on later stages (Angilletta, 2009). Acclimation and developmental plasticity are linked and may interact to shape an individual’s ability to respond to environmental change during its lifetime (Beaman *et al.*, 2016).

The response of organisms to stressor exposure is most commonly studied during the exposure to evaluate the effects of the stressor; however, there are potentially carry-over effects from the stressor after the stressor is removed that are often not measured. Carry-over effects occur when an individual’s prior history or experience influences their performance in a given situation, often after a discrete temporal, seasonal or developmental transition (O’Connor *et al.*, 2014). Carry-over effects can be persistent, where they occur during the exposure and remain present when the stressor is removed, or latent, where effects of the exposure appear only in later life stages after the stressor is removed (Pechenik, 2006). Through carry-over effects, the influence of earlier developmental stressors can be observed in the phenotypes of juvenile or adult life stages, even when the stressors are no longer present. Carry-over effects in response to warming (Hanson *et al.*, 2008; Macqueen *et al.*, 2008; Scott and Johnston, 2012; Campos *et al.*, 2013; Schnurr *et al.*, 2014) or hypoxia (Johnston *et al.*, 2013; Robertson *et al.*, 2014; Vanderplancke *et al.*, 2015; Cadiz *et al.*, 2017; Zambonino-Infante *et al.*, 2017) experienced during early fish development have both been investigated as individual stressors in numerous fish species (Vagner *et al.*, 2019). In contrast, very few studies have focused on the persistent, carry-over effects of multiple stressors following developmental exposure, particularly in fishes (Zambonino-Infante *et al.*, 2013).

In California, at the southern end of the range for Chinook salmon, *Oncorhynchus tshawytscha*, the progression of global climate change combined with periods of prolonged drought can increase water temperatures, reduce water flows and decrease DO (Hanak *et al.*, 2015). Chinook salmon populations in central California are among the most vulnerable within the species (Crozier *et al.*, 2019) and may serve as a model system to predict how climate change can affect species at the southern end of their distributions as they are already experiencing multiple stressors. Chinook salmon embryos incubating within redds (or gravel nests) are the most thermally sensitive life stage, have no ability to swim away from suboptimal conditions and occupy freshwater habitats that differ markedly from later estuarine and marine stages (Myrick and Cech, 2004; Williams, 2006). For anadromous species such as salmon, developmental plasticity could affect the migratory ability of early life stages or influence acclimation capacity and stress tolerance during later life stages that outmigrate through diverse and dynamic habitats to the ocean. Conservation measures to support vulnerable fish species may benefit from a stage-specific approach, particularly when habitats and abiotic conditions vary with ontogeny (Komoroske *et al.*, 2014). Understanding how salmon embryos respond to stressors during different developmental stages and how early exposure can affect the physiological performance of later life stages can inform stage-specific management strategies, such as determining when releases of limited cold water resources are most needed by developing salmon in tributaries of the Sacramento and San Joaquin basins with competing societal and environmental water needs in the San Francisco Bay Delta.

In this study, we investigated the acute and persistent effects of early developmental exposure to multiple stressors across four developmental stages in Chinook salmon. To examine the effects of stressor type and how the timing of stressor exposure affected physiological performance of Chinook salmon, we compared the physiological responses of embryos at two stages to a warm temperature, hypoxia or both stressors during chronic and short term-exposures. To study carry-over effects of the embryonic rearing environment on the physiology, growth and development of later life stages, after hatching all fish were transferred to control conditions and tested at two post-hatch stages. We predicted that (i) the multiple stressor treatments of warm temperature and hypoxia would have the greatest effects on salmon development during the embryonic exposures as well as during later life stages, (ii) salmon exposed to stressors chronically or late during embryonic development would experience more detrimental effects due to increasing energy demand as development progresses and (iii) carry-over effects would be strongest in fish reared under chronic embryonic stress because of the impact of exposure to stressors during the longer term development of many physiological systems.

## Materials and methods

### Fish acquisition and care

Freshly fertilized late-fall-run Chinook salmon embryos were obtained from six breeding pairs spawned at the Coleman National Fish Hatchery (US Fish and Wildlife Service, Anderson, CA). Embryos were transported to the University of California Davis Center for Aquatic Biology and Aquaculture in January 2019 and immediately transferred to their rearing treatments in one of three replicate 15-l square culture buckets. Each replicate contained 111 embryos reared in floating mesh baskets affixed with a plastic grid creating individual wells to keep embryos separated in an even layer. Embryos from the six families were evenly distributed across each replicate bucket. The baskets were removed from the culture buckets when the alevins could sustain swimming. The fish were not fed during the experiment since early developmental stages rely on endogenous yolk reserves. The experiment ended when fish reached the fry stage and the yolk sac was almost completely absorbed. All fish care and protocols were approved by the UC Davis Institutional Animal Care and Use Committee (protocol no. 19593).

### Experimental design

**Figure 1:**
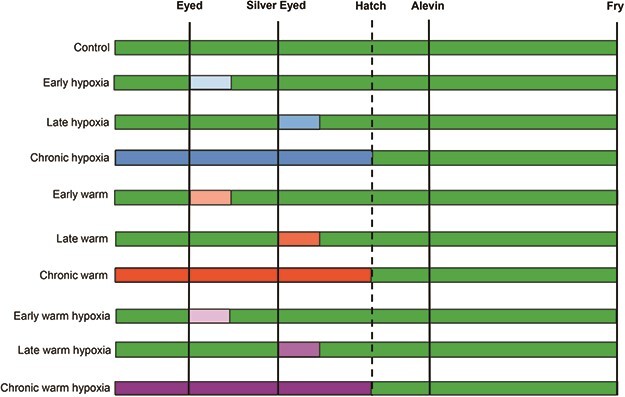
Developmental windows of exposure to hypoxia (50% DO, 10°C, blue bars), warm temperature (100% DO, 14°C), red bars, or warm temperature and hypoxia (50% DO, 14°C, purple bars). Control conditions (100% DO, 10°C) are represented by green bars, and sampling time points are indicated by solid vertical lines. Following hatch, all treatments were transferred to control conditions.

To assess the effects of elevated temperature and decreased oxygen as individual and combined stressors at different stages in embryonic development, we reared developing Chinook salmon from fertilization to the fry stage in 10 treatments with two temperatures [10°C (ambient) and 14°C (warm)], two DO saturation levels [normoxia (100% air saturation, 10 mg O_2_/l) and hypoxia (50% saturation, 5.2–5.7 mg O_2_/l)] and three exposure times (early [eyed stage, when dark pigmented eyes are visible], late [silver-eyed stage, when silver pigmented irises are visible] and chronic [fertilization through hatching]) (Fig. 1, see Supplementary Material Table 1).

**Table 1:** Developmental timing of each treatment with the number of days post-fertilization it took to reach each stage: eyed stage, when dark pigmented eyes were clearly visible; silver-eyed stage, when a silver pigmented iris was visible; hatch, when 90% of the embryos in a treatment hatched; and the fry stage, when 75% of fish in a treatment had absorbed nearly all of their yolk sac.

Treatment	Eyed stage	Silver-eyed stage	Hatch	Fry stage
Control	25	34	51	85
Early hypoxia	25	35	54	89
Late hypoxia	25	34	52	88
Chronic hypoxia	28	37	53	94
Early warming	25	31	48	83
Late warming	25	34	49	82
Chronic warming	17	22	34	73
Early warm hypoxia	25	32	50	84
Late warm hypoxia	25	34	55	87
Chronic warm hypoxia	20	26	37	81

Due to the equal mix of families among replicates, fish across replicates with the same temperature and oxygen conditions consistently reached each stage on the same day such that there was no variability in developmental timing within a treatment.

The temperatures and DO levels were chosen and maintained for three replicate culture buckets per treatment (temperature x oxygen x stage combination), as described in Del Rio *et al.*, (2019). Briefly, the temperatures of 10°C and 14°C represent a water temperature that supports salmon embryo survival in central California and a 4°C increase in water temperature predicted to become more common due to climate change, respectively. The hypoxic value of 50% air saturation was chosen as DO within redds can be highly variable and regularly drops below 50% due to low hyporheic flows and oxygen consumption by embryos as water flows through a redd (Peterson and Quinn 1996; Martin *et al.*, 2020). Temperatures were maintained by placing the culture buckets in water bath tanks and DO levels were individually controlled in each bucket by dripping in fresh water from a reservoir mixed with air (normoxia) or N_2_ gas (hypoxia) at 4 l/h and with air stones that bubbled in air (normoxia) or N_2_ gas (hypoxia). Temperature and DO were measured in each culture bucket daily using a handheld OxyGuard Handy Polaris 2 metre (OxyGuard International, Farum, Denmark). To assess stage-specific sensitivity to stressors during embryonic development, the exposures to a warm temperature and hypoxia occurred either chronically, from the day of fertilization through hatching, early, for 5 days upon reaching the eyed embryo stage, or late, for 5 days upon reaching the silver-eyed embryo stage. The exposure time of 5 days allowed time to adjust to the stressor(s) and avoided confounding effects of hatching because the shortest time between the silver-eyed stage and hatching was estimated to be 6 days (Del Rio *et al.*, 2019). When embryos in early or late warm treatments experienced a short-term warm temperature exposure, the water bath tank was increased from 10°C to 12°C over 6 h and then from 12°C to 14°C to reach the 14°C target temperature within 12 h. Following 5 days at 14°C, the temperature on the sixth day after the exposure began was decreased within the water bath from 14°C to 12°C over 6 h and then decreased to 10°C for the remainder of the experiment. When treatments experienced early or late hypoxia the water source was transferred to a hypoxia reservoir bucket, which decreased DO to 80% over 6 h before nitrogen was bubbled in via air stones to decrease DO to the final hypoxic level of 50% air saturation within 12 h and held for the remainder of the 5 days. On the sixth day after the hypoxia exposure began, nitrogen bubbling was reduced and buckets were switched to a normoxic reservoir water source until DO reached 80% saturation over 6 h and then air was bubbled in to reach 100% saturation over 6 h and held for the remainder of the experiment. When the chronic treatments were 90% hatched or more, they were switched to control conditions of 10°C normoxic water following the incremental procedures used for the short-term exposures.

Physiological testing occurred twice for each treatment during the embryonic stages following a stage-based sampling design to account for differences in developmental rate caused by the varying temperatures and DO levels between treatments. Sampling took place when 75% or more of embryos in a treatment reached (i) eyed stage, when dark pigmented eyes were clearly visible and (ii) silver-eyed stage, when a silver pigmented iris was visible. Salmon embryo development was monitored daily with visual inspections of each culture bucket. Stage was assessed at the treatment level since there was little variation in developmental timing between replicates, likely because embryos from each family were equally distributed among replicates. Hatching success was calculated as the ratio between the number of alevins hatched and the initial number of embryos per treatment (*n* = 333 per treatment). Routine metabolic rate (RMR) was assessed at both stages (eyed, silver-eyed).

### Embryo respirometry

RMR of embryos was measured using closed system respirometry at two embryonic stages. In all treatments RMR was measured 5 days after reaching each embryonic stage to capture the metabolic response on the last day of exposure for short-term exposure treatments and the metabolic rate of embryos in chronic or control conditions at a comparable developmental stage. A glass microplate (Loligo Systems, Viborg, Denmark) containing 24, 1.7-ml wells, each with an O_2_ sensor spot, was loaded with 18 embryos per treatment (3 embryos per family, 6 per replicate) and sealed with a butyl rubber gasket. Six wells were left empty to measure background respiration, which was minimal during all trials. A water bath surrounding the microplate maintained the water temperature at the rearing or exposure temperature each treatment was held in at that stage (10°C or 14°C). The sealed microplate and water bath were placed on an O_2_ fluorescence sensor (SDR SensorDish Reader, PreSens, Regensburg, Germany), which sat on a laboratory rocker to create gentle mixing within each well. DO in the microplate started at the concentration of the rearing treatment, so fish in normoxia started at 100% air saturation and fish in hypoxia started at 50% (Miller *et al.*, 2008). Metabolic rate was analysed for the first 20% drop in oxygen saturation (i.e. until saturation reached 80% in normoxia and 30% in hypoxia) (Miller *et al.,* 2008). At the end of each trial, the embryos were removed from the microplate, euthanized with a lethal dose of buffered tricaine methanesulfonate (MS-222, 500 mg·l^−1^, Western Chemical, Ferndale, WA, USA), blotted dry and weighed whole.

### Carry-over effects of developmental exposure

To evaluate whether stressors experienced during embryonic development could have lasting effects on subsequent life stages when the stressors are no longer present, each treatment was reared in control conditions of 10°C and 100% air saturation following the embryonic exposure. All treatments were held under control conditions through the fry stage and were sampled at the alevin stage (5 days post-hatch) and the fry stage, when the yolk sac was almost completely absorbed. The proportion of dry body tissue mass to dry total mass was measured at the alevin stage as a metric of size shortly post-hatch. RMR was assessed at both post-hatch stages (alevin and fry). Critical thermal maximum (CTMax) and hypoxia tolerance were tested at the fry stage only. Wet mass and total length were also measured at the fry stage to calculate Fulton’s condition factor.

### Alevin and fry respirometry

RMR of alevins and fry was measured using automated intermittent flow through respirometry. Fish (*n* = 16 per treatment) were placed in individual glass respirometry chambers held in a water bath at 10°C and 100% air saturation. Each chamber (7.7 ml for alevins, 20.7 ml for fry) contained an oxygen sensor spot that was read by a fibre optic oxygen metre to record the decrease in oxygen over five measurement cycles (Witrox 4, Loligo Systems, Denmark). Each cycle consisted of a 15-min flush period, a 3-min wait period and a 12-min measurement period. During measurement periods peristaltic pumps recirculated water through the chamber in a closed circuit to ensure even mixing. Oxygen did not decrease below 80% air saturation during measurement periods. During flush periods peristaltic pumps circulated fresh, fully oxygenated water from the surrounding water bath through the chambers and into a waste water collection container to reoxygenate the chambers and remove waste products before the start of the next measurement period. Following each set of trials, oxygen consumption within a blank chamber was run for one measurement period to calculate background respiration. After the RMR trials the fish were euthanized in buffered MS-222 (500 mg·l^−1^) and measured for total length and weight. The first two measurement periods were excluded from the analysis for all trials as the fish were likely recovering from handling stress, which can increase metabolic rate. Therefore, we measured RMR as the average of the last three measurement periods of each trial when fish were quiescent.

### Fry upper thermal tolerance

Acute upper thermal tolerance was measured using critical thermal maximum (CTMax) methodology (Beitinger *et al.,* 2000; Fangue *et al.,* 2006) as described in Del Rio *et al.* (2019). CTMax was determined for 5–6 fish per replicate per treatment (*n* = 16 fish per treatment). Individual fish were placed in the jars for 1 h prior to the start of each trial with water at 10°C and air continuously bubbled into each jar. After the 1 h acclimation a heater was turned on and the water temperature increased at a rate of 0.3°C/min. Fish were closely monitored until they reached loss of equilibrium (LOE), defined as the point at which a fish could no longer swim upright or respond to a gentle physical stimulus. Temperature at LOE was recorded with a calibrated immersion thermometer (0.1°C precision, Fisher Scientific), after which individuals were immediately transferred to a fully oxygenated recovery tank at 10°C. Temperature at LOE was included in the final dataset if the individual survived a 24-h recovery period.

### Fry hypoxia tolerance

Acute hypoxia tolerance of salmon fry was measured using time to LOE methodology following Del Rio *et al.* (2019). Time to LOE was determined for 5–6 fish per replicate per treatment (*n* = 16 fish per treatment). Hypoxia tolerance trials were conducted at 10°C in an aquarium containing 16 floating plastic beakers. Following a 1-h recovery period after fish were placed in individual beakers, DO of the water was reduced at a rate of 1.5–2%/min by bubbling in N_2_ gas until 7% air saturation was reached (0.8 mg/l). Oxygen was then held at 7% by manually adjusting the flow of N_2_. This final oxygen concentration was chosen based on pilot studies where there was little variation in the rapid time to LOE at 6%. Time to LOE was defined as the time (min) after DO saturation reached 7% until the fish could no longer swim upright or respond to a gentle physical stimulus. Upon achieving LOE fish were immediately transferred to fully oxygenated recovery chambers at 10°C. Time to LOE for fish that survived a 24-h recovery period was included in the final dataset. Fish that maintained equilibrium when the 2-h trial ended were assigned a time to LOE of 120 min and transferred to recovery.

### Alevin size

Following respirometry trials at the alevin stage, individuals were euthanized in buffered MS-222 (500 mg·l^−1^). The yolk and body tissues were separated and placed in individual pre-weighed aluminium weigh boats. Tissues were dried in an oven at 65°C for 48 h. The dried tissues were weighed and the proportion of dry body tissue to total dry mass was calculated as

$$ \frac{\mathrm{dry}\ \mathrm{body}\ \mathrm{mass}}{\left(\mathrm{dry}\ \mathrm{body}\ \mathrm{mass}+\mathrm{dry}\ \mathrm{yolk}\ \mathrm{mass}\right).} $$

### Fry condition factor

Following respirometry, CTMax and hypoxia trials, fish at the fry stage (*n* = 16 per replicate, *n* = 48 per treatment) were euthanized in buffered MS-222 (500 mg·l^−1^), weighed and measured for total length. Body condition was used to compare overall size differences between treatments. Fulton’s condition factor (K) was calculated as

$$ K=100\ x\ \frac{W}{L^3}, $$

where *W* is the wet mass in grams and *L* is the total length of the fish in cm.

### Statistical analyses

All statistical analyses were performed in R with the R Studio interface (v3.6.3, R Core Team, 2020). Data sets were inspected for assumptions of normality and homoscedasticity visually using Q-Q plots and residuals vs. fitted values as well as Shapiro–Wilkes and Levene’s tests. Datasets that did not meet these assumptions were either log or cube root transformed. Treatment was tested as a fixed main effect using one-way analysis of variance (ANOVA) and differences between treatments were assessed using Tukey’s post hoc tests and the ‘lsmeans’ package (Lenth, 2016). The individual effects of stressor type (warm temperature, hypoxia or both stressors) and stage of exposure (early, late, or chronic) were compared relative to the control conditions using contrasts and the ‘lsmeans’ package with a Sidak correction for multiple comparisons. Hatching success was analysed using a generalized linear mixed model and the ‘lme4’ package (Bates *et al.*, 2015). Treatment was a fixed main effect and replicate was included as a random effect, with a binomial error distribution. The remaining datasets were analysed with linear models or linear mixed effects models using the base ‘stats’ package as well as the ‘lme4’ and ‘car’ packages (Fox and Weisberg, 2011). Respirometry data was first visually inspected and abnormal or poor quality traces were excluded. Data points were excluded from embryo RMR datasets if the r^2^ was below 0.75, indicating low quality of the traces. Data points in the post-hatch RMR datasets had an r^2^ of 0.85 or higher. Experimental chamber was included as a random effect in models for alevin and fry RMR. Data are reported as means ± SEM with α set to 0.05.

## Results

### Hatching success

**Figure 2:**
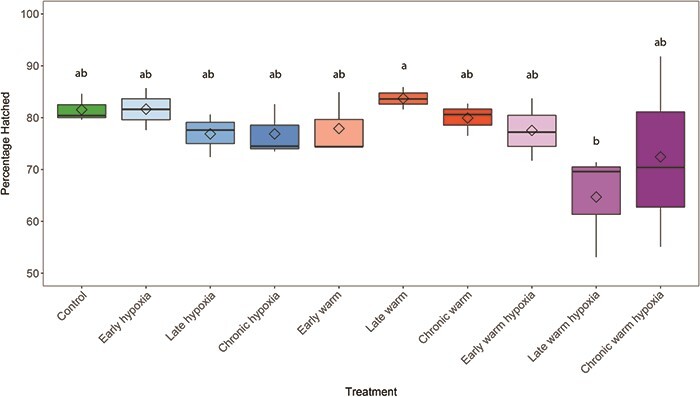
Percentage of embryos hatched (*n* = 3 per treatment). Boxplots of the data represent the median as the centre line, interquartile range (IQR) as the box, values 1.5 times the IQR as the whiskers and values greater than 1.5 times the IQR as black points. Diamonds indicate the mean value. Colours represent the developmental treatment: control (green, 100% DO, 10°C), hypoxia (blue, 50% DO, 10°C), warm (red, 100% DO, 14°C) or warm hypoxia (purple, 50% DO, 14°C). The three boxplots within each stressor type represent the developmental timing of exposure as early (eyed stage, light colour shade), late (silver-eyed stage, medium colour shade) and chronic (fertilization through hatching, dark colour shade). Letters indicate significant differences among treatments (*P* < 0.05).

Treatment had a significant effect on hatching success (Wald χ^2^ = 19.21, *P* = 0.023) (Fig. 2). The late warm and late warm hypoxia treatments were significantly different from each other, with late warm temperature resulting in the highest hatching success (83.7% ± 1.2) and late warm hypoxia resulting in the lowest hatching success (64.7% ± 5.8). Overall, there were no significant effects of warming (*P* = 0.99), oxygen (*P* = 0.96) or combined stressors (*P* = 0.14) either early (*P* = 0.98), late (*P* = 0.63) or chronically (*P* = 0.78) on hatching success relative to the control treatment (81.5% ± 1.55).

### Alevin size

**Figure 3:**
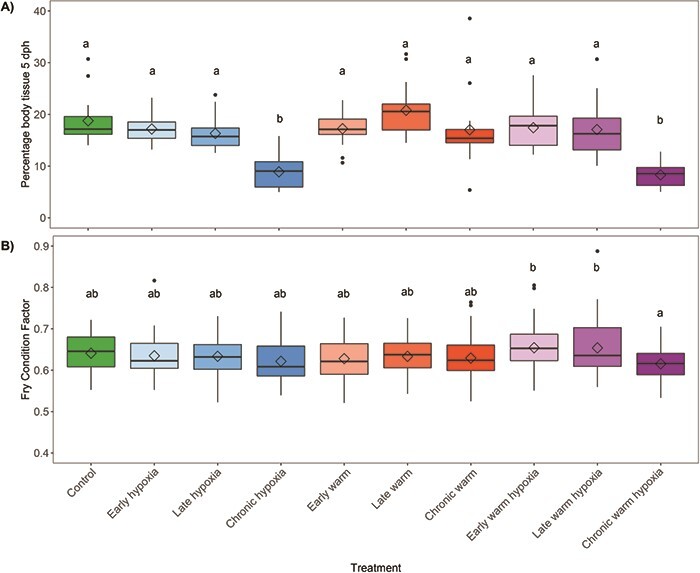
Post-hatch size at the A) alevin stage as the percentage of dry body tissue mass relative to total yolk and body dry mass of alevins 5 days post hatch (*n* = 16 per treatment except chronic normoxia [*n* = 15] and chronic warm hypoxia [*n* = 13]) and B) fry stage as Fulton’s condition factor (*n* = 47 per treatment except late hypoxia and late warm hypoxia [*n* = 45], early hypoxia and chronic warming [*n* = 46] and early warming and early warm hypoxia [*n* = 48]). Boxplots of the data represent the median as the centre line, IQR as the box, values 1.5 times the IQR as the whiskers and values greater than 1.5 times the IQR as black points. Diamonds indicate the mean value. Colours represent the developmental treatment: control (green, 100% DO, 10°C), hypoxia (blue, 50% DO, 10°C), warm (red, 100% DO, 14°C) or warm hypoxia (purple, 50% DO, 14°C). The three boxplots within each stressor type represent the developmental timing of exposure as early (eyed stage, light colour shade), late (silver-eyed stage, medium colour shade) and chronic (fertilization through hatching, dark colour shade). Letters indicate significant differences among treatments (*P* < 0.05).

The percentage of body tissue mass relative to total mass (body + yolk) differed significantly among treatments 5 days post-hatch (*F*_9,154_ = 19.08, *P* < 0.001) (Fig. 3A). Alevins in the chronic hypoxia and chronic warm hypoxia treatments were significantly smaller compared to the other treatments. Compared to control conditions, the chronic exposure time (*P* < 0.001), hypoxia (*P* < 0.001) and warm hypoxia stressors (*P* < 0.001) were significant factors affecting alevin size post-hatch.

### Fry condition factor

Condition factor was significantly different among treatments at the fry stage (*F*_9,456_ = 2.84, *P* = 0.003). No treatments were significantly different from the control (Fig. 3B); however, the chronic warm hypoxia treatment had the lowest condition factor and was significantly different from the early and late warm hypoxia treatments, which had the highest condition factors. The type of stressor (warm temperature, hypoxia or both) and stage of exposure (early, late or chronic) were not significant (*P* > 0.05).

### Embryo RMR

#### Eyed stage

**Figure 4:**
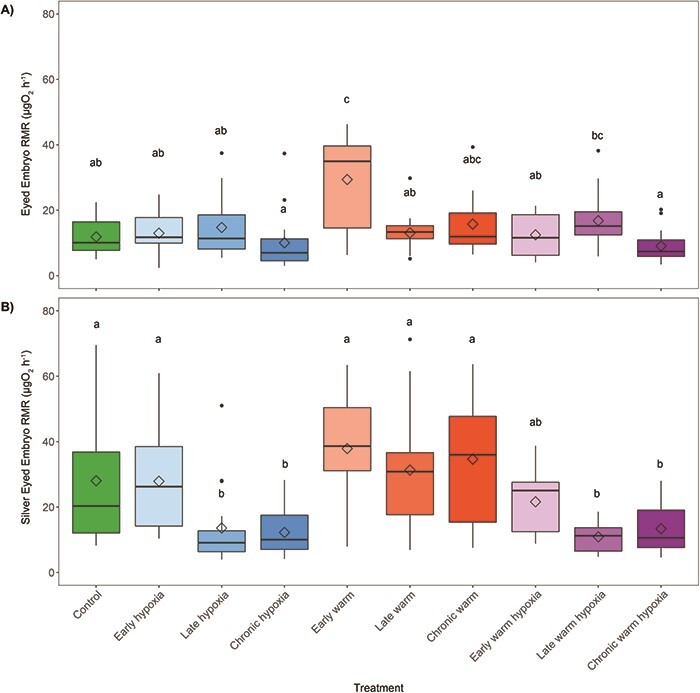
RMR in μgO_2_ h^−1^ per embryo per treatment at the A) eyed stage (control *n* = 18, late hypoxia, late warm, late warm hypoxia and chronic warm hypoxia [n = 17], early hypoxia and early warm [*n* = 16], chronic warm [*n* = 15] and early warm hypoxia [n = 14]) and B) silver-eyed stage (*n* = 18 per treatment except late hypoxia, late warm and chronic warm hypoxia [n = 17] and control and early hypoxia [n = 16]). Boxplots of the data represent the median as the centre line, IQR as the box, values 1.5 times the IQR as the whiskers and values greater than 1.5 times the IQR as black points. Diamonds indicate the mean value. Boxplot fill colours represent the developmental treatment: control (green, 100% DO, 10°C), hypoxia (blue, 50% DO, 10°C), warm (red, 100% DO, 14°C) or warm hypoxia (purple, 50% DO, 14°C). The three boxplots for each stressor type represent the developmental timing of exposure as early (eyed stage, light colour shade), late (silver-eyed stage, medium colour shade) and chronic (fertilization through hatching, dark colour shade).

There were significant differences in RMR among treatments 5 days after reaching the eyed embryo stage (*F*_9,153_ = 5.46, *P* < 0.001) (Fig. 4A). At this stage, the early and chronic treatments were experiencing one or both stressors and the late treatments were still under control conditions (Fig. 1). The early warm treatment had the highest RMR, which was significantly higher than the control, chronic and late hypoxia, chronic and early warm hypoxia and late hypoxia treatments. The type of stressor (warm temperature, hypoxia or both) or stage of exposure (early, late or chronic) did not individually affect RMR relative to control conditions. Family was not a significant factor and was excluded from the statistical analysis (see Supplementary Material, Fig. S1).

#### Silver-eyed stage

Five days after the silver-eyed stage there were significant differences in RMR due to treatment (*F*_9,161_ = 11.32, *P* < 0.001) (Fig. 4B). At this stage, the late and chronic treatments were exposed to one or both stressors and the early treatments had returned to control conditions (Fig. 1). The early warm treatment continued to have the highest RMR and was significantly higher than the early, late and chronic warm and hypoxia and late and chronic hypoxia treatments. The RMR of the late and chronic warm and hypoxia treatments were significantly lower than the control treatment. There was a significant effect of hypoxia (*P* = 0.03), the multiple stressors (*P* = 0.007) and the late exposure stage (*P* = 0.04) relative to control conditions. Family was not a significant factor and was excluded from the statistical analysis (Supplementary Material, Fig. S2).

### Alevin RMR

**Figure 5:**
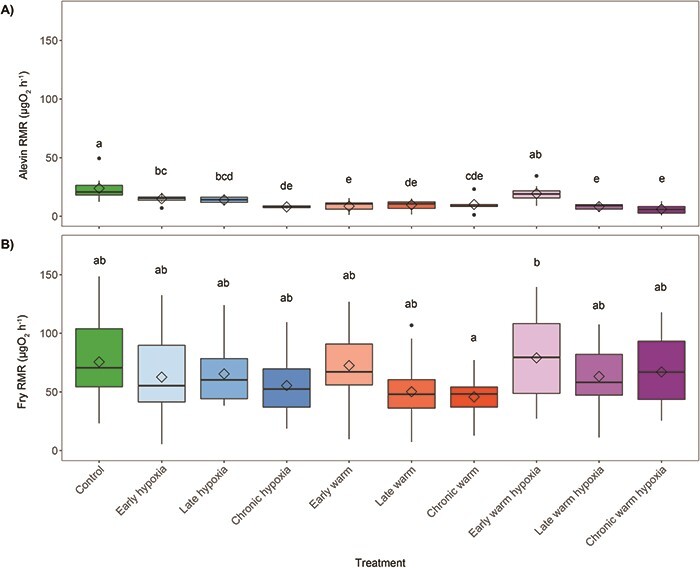
Post-hatch RMR in μgO_2_h^−1^ per individual at the A) alevin stage 5 days post-hatch (*n* = 16 per treatment except early warm [*n* = 15], control [*n* = 11], chronic warm [*n* = 6] and chronic hypoxia and chronic warm hypoxia [*n* = 5]) and B) fry stage (*n* = 16 per treatment except for late hypoxia and chronic warm [*n* = 15]). Boxplots of the data represent the median as the centre line, IQR as the box, values 1.5 times the IQR as the whiskers and values greater than 1.5 times the IQR as black points. Diamonds indicate the mean value. Colours represent the developmental treatment: control (green, 100% DO, 10°C), hypoxia (blue, 50% DO, 10°C), warm (red, 100% DO, 14°C) or warm hypoxia (purple, 50% DO, 14°C). The three boxplots for each stressor type represent the developmental timing of exposure as early (eyed stage, light colour shade), late (silver-eyed stage, medium colour shade) and chronic (fertilization through hatching, dark colour shade). Letters indicate significant differences between treatments (*P* < 0.05).

Alevin RMR was significantly affected by developmental treatment (Wald χ^2^ = 142.55, *P* < 0.001). After a minimum of 5 days in control conditions following hatch, all treatments except the early warm hypoxia treatment had a significantly lower RMR than the control treatment (Fig. 5A). Additionally, all three stages of exposure (early, late, chronic) and each type of stressor (warm temperature, hypoxia, warm temperature and hypoxia) were significantly different from control conditions (*P* < 0.05). The low RMR of the alevins compared to the silver-eyed stage embryos may be due to the use of intermittent flow respirometry at the alevin stage, which allows for an acclimation period, and closed respirometry for embryos that did not allow for acclimation after handling.

### Fry RMR

Developmental stress treatment significantly affected the RMR of salmon at the fry stage (Wald χ^2^ = 21.56, *P* = 0.01). No treatments were significantly different from the control treatment, but the chronic warm treatment had the lowest RMR and was significantly different from the early warm hypoxia treatment, which had the highest RMR (Fig. 5B). There were no significant effects of stressor type or stage on RMR relative to control conditions.

### Upper temperature tolerance (CTMax)

**Figure 6:**
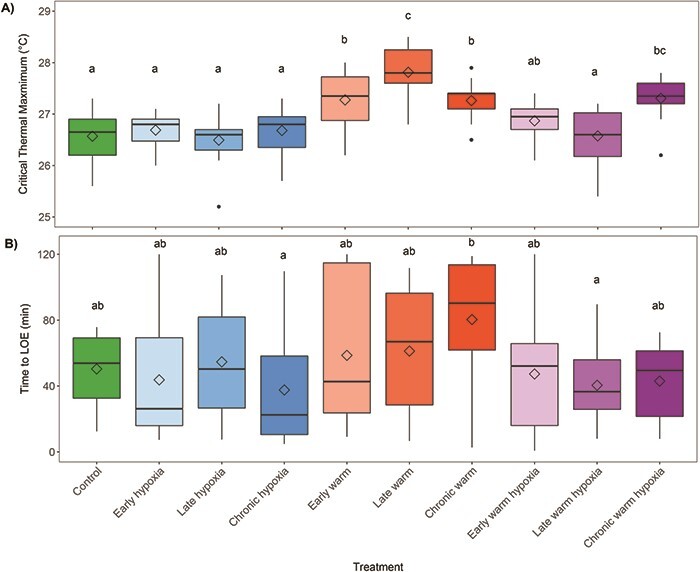
Acute thermal and hypoxia tolerance at the fry stage measured as A) critical thermal maximum (°C) (*n* = 16 per treatment except for late hypoxia, chronic hypoxia and late warm [*n* = 15]) and B) time to LOE (minutes) in hypoxia (*n* = 16 per treatment except for early hypoxia [*n* = 15]). Boxplots represent the median as the centre line, IQR as the box, values 1.5 times the IQR as the whiskers and values greater than 1.5 times the IQR as black points. Diamonds indicate the mean values. Colours represent the developmental treatment: control (green, 100% DO, 10°C), hypoxia (blue, 50% DO, 10°C), warm (red, 100% DO, 14°C) or warm hypoxia (purple, 50% DO, 14°C). The three boxplots for each stressor type represent the developmental timing of exposure as early (eyed stage, light color shade), late (silver-eyed stage, medium color shade) and chronic (fertilization through hatching, dark color shade). Letters indicate significant differences among treatments (*P* < 0.05).

Developmental rearing conditions significantly affected upper temperature tolerance, as measured by CTMax, at the fry stage (*F*_9,147_ = 14.47, *P* < 0.001) (Fig. 6A). The late warm treatment had the highest CTMax (27.8°C ± 0.13) while early warming (27.3°C ± 0.14), chronic warm (27.3 ± 0.10) and chronic warm hypoxia (27.3°C ± 0.10) treatments also had a significantly higher CTMax than the control treatment. The remaining treatments did not differ significantly and were very similar to the control treatment (26.5°C ± 0.11). There were significant effects of warm temperature (*P* < 0.001), multiple stressors (*P* = 0.05) and early (*P* = 0.02), late (*P* = 0.02) and chronic (*P* < 0.01) exposure on CTMax, relative to control.

### Hypoxia tolerance

Developmental stress exposure significantly affected hypoxia tolerance at the fry stage among treatments (*F*_9,149_ = 2.20, *P* = 0.03) (Fig. 6B). While no treatments were significantly different from the hypoxia tolerance (47.4 min ± 6.06) of salmon reared under control conditions, the chronic warm treatment had the highest hypoxia tolerance (80.4 min ± 10.27) and was significantly more tolerant than the chronic hypoxia (37.6 min ± 8.2) and late warm hypoxia treatments, which had the lowest hypoxia tolerance (37.6 min ± 8.2 and 40.5 min ±5.8, respectively). There was no significant effect of the stressor type or stage of exposure on hypoxia tolerance relative to control conditions.

### Developmental timing

Developmental rate was assessed at the treatment level because there was very little variation in developmental timing across replicate buckets within the same treatment. Warm temperature, hypoxia or both stressors influenced the rate of development during both short and chronic exposures (Table 1). The warmer temperature increased the rate of development, with the chronic warm treatment hatching in the shortest amount of time, 34 days post-fertilization (dpf). Hypoxia delayed development, with the early and chronic hypoxia treatments taking the longest amount of time to hatch (53–54 dpf). The effects of developmental exposure to one or both stressors led to differences in the amount of time it took for each treatment to reach the fry stage even though all treatments were raised in control conditions after hatching.

## Discussion

In this study we investigated how warmer water temperature, hypoxia or both warm temperature and hypoxia affected the physiology and development of Chinook salmon embryos and larvae. We tested the effects of short and chronic exposures to evaluate the sensitivity of developmental stages both during and after stressor exposures to study when during embryonic development embryos were most sensitive to environmental stressors as well as the potential for stressor exposures of different durations to have carry-over effects post hatch. Overall, rearing salmon embryos under warm water temperature and warm temperature with hypoxia conditions had the greatest effects on RMR, both during exposure and in later life stages, after the stressors were removed; however, the effects of these stressors varied across development. Developmental exposure to the warmer water temperature also had the largest effects on acute stress tolerance at the fry stage. Consistent with our hypotheses, the developmental exposures experienced chronically or at a late embryonic stage had the strongest effects on the physiology of post-hatch stages.

### Hatching success

Warm temperature and hypoxia did not reduce hatching success as individual stressors, indicating the temperature of 14°C and DO level of 50% are not lethal for this population of Chinook salmon embryos. However, the combination of stressors, experienced late in embryonic development, resulted in the lowest hatching success. The majority of mortality occurred later in development, between the silver-eyed stage and hatching (see Supplementary Material Fig. S3). The late embryonic stage may be more sensitive to stressors because oxygen consumption increases steadily as salmon embryos develop and critical oxygen tensions peak at the time of hatching (Rombough, 1988b; Wood *et al.*, 2019). Experiencing the combination of warm temperature and hypoxia that both increases oxygen demand and reduces supply at a stage where oxygen consumption and sensitivity is already high appears to be particularly detrimental to hatching. The process of hatching also requires additional energy and can increase oxygen consumption by 19% (Ninness *et al.*, 2006). This increased sensitivity to stressors late in embryonic development may support management strategies that aim to prioritize cold water releases, when cold water availability is limited, for salmon embryos shortly before hatch to use available water when it will be most effective for minimizing hatching failure. Although hatching occurs over several weeks due to differences in spawn timing, stage-specific water releases may still conserve water by maintaining cold water over a 3-month period instead of the 5 months proposed to capture the majority of the time salmon embryos are present (James Anderson, pers. comm.).

### Growth and development

Growth is a highly energy-intensive process during early fish development (Rombough, 2011). At the alevin stage, 5 days post-hatch, salmon reared under the chronic hypoxia and chronic warm hypoxia treatments were smaller, with a lower proportion of body tissue mass relative to total mass and therefore had more yolk mass. Reduced size at hatch is a common response to rearing in hypoxia (Alderdice *et al.*, 1958; Garside, 1959), thought to result from molecular responses that conserve ATP by downregulating genes for cell growth and protein synthesis (Gracey *et al.*, 2001; Ton *et al.*, 2003). Salmon reared under chronic hypoxia had significantly lower RMR relative to other treatments during embryonic development and this could have resulted in less energy being allocated to growth. Fish that are smaller in size can be more vulnerable to predation or have reduced swimming and competitive abilities (Mason, 1969; Pepin, 1991), which can further increase the risk for early life stages that are already prone to high predation and mortality (Kamler, 1992). Upon reaching the fry stage (32–44 days post hatch), condition factor was similar among treatments as well as length and mass (see Supplementary Material, Figs S4 and S5). The fish were not fed during the experiment so similarities in condition factor may reflect similar utilization of yolk reserves during development, although this was not tested. However, the amount of time it took each treatment to reach the fry stage after hatching ranged from 32–44 days. The developmental rate of fish embryos is known to be highly dependent on temperature and DO in the incubation environment (Murray and McPhail, 1988; Beacham and Murray, 1990). Here, we show that the developmental rate of post hatch stages continues to be influenced by the temperature and DO levels experienced during embryonic development, even though the larvae are no longer being exposed to these conditions. Although fish in the chronic warm and chronic warm hypoxia treatments developed the fastest during embryonic exposure hatching 34 and 37 dpf compared to 51 dpf in the control treatment, their development was delayed when transferred to control conditions post hatch as the chronic warm treatment reached the fry stage 39 days post hatch (dph) and the chronic warm hypoxia treatment after 44 dph compared to 34 dph in the control group (Table 1). Both growth and developmental timing are important in the life cycle of salmonids and can impact the survival of juvenile stages though adjustments to migration phenology (Jonsson *et al.*, 2017). Therefore, changes in developmental rate and growth could affect salmon in the Central Valley as they begin migrating to the predator dense San Francisco Bay Delta shortly after reaching the fry stage, a 445-km journey for fish from the Coleman National Fish Hatchery (Sturrock *et al.,* 2019). It would be important to investigate whether these changes in developmental timing affected the timing and process of smoltification as well, which could further delay outmigration (Munsch *et al.*, 2019).

### Embryo **RMR**

In this study, eyed embryos exposed to early warm water temperature had a higher RMR, while the chronic warm treatment RMR did not differ from the control treatment. This suggests that eyed stage embryos can adjust their metabolic rate if given enough time in the exposure (i.e. 22 days in the current experiment), but that a short-term exposure of 5 days was not sufficient for metabolic adjustment. Dragonfish embryos were found to be more thermally sensitive early in development at the gastrulation stage compared to later embryonic stages evidenced by a higher temperature coefficient (Q_10_) (Flynn and Todgham, 2018). Stage-specific differences in thermal physiology such as temperature sensitivity of biological and biochemical processes may contribute to the stronger effects of warm temperature at the eyed stage. The early warm treatment continued to have the highest RMR at the silver-eyed stage, even after being returned to control temperatures for 6 days prior to measurement, demonstrating persistent effects of early warm temperature on RMR. Temperature increases and decreases from initial incubation temperatures, early in the embryonic development of lake whitefish, also resulted in persistent changes to metabolic rate, particularly when temperature was shifted between the gastrulation and organogenesis stages (Eme *et al.*, 2015).

Metabolic suppression is a common response to hypoxia exposure, especially as environmental oxygen concentrations drop below critical oxygen tension levels (Richards, 2009). Although the mechanisms underlying RMR reductions in fish embryos are not fully understood, it is thought that embryos have a limited capacity for anaerobic metabolism and therefore rely on metabolic suppression to cope with hypoxia (Ninness et al., 2006; Rombough, 2011). Reduced RMR in hypoxia relative to normoxia-reared embryos has been observed in several other studies (Hamor and Garside, 1979; Miller *et al.*, 2008; Wood *et al.*, 2019). Differences in metabolic responses to chronic and short term hypoxia are dependent on developmental stage and exposure duration as physiological thresholds and responses vary across embryonic development (Miller *et al.*, 2008). Here, the RMR of eyed stage embryos exposed to early or chronic hypoxia was not significantly different from the control treatment, potentially because oxygen demands are relatively low at this stage. In contrast, the late and chronic hypoxia and multiple stressor treatments had a lower RMR at the silver-eyed stage. Under normoxic conditions, oxygen consumption and critical oxygen tensions (Pcrit) are higher at later stages of embryogenesis (Rombough, 1988b). Pcrit for the eyed stage embryos was likely near or below the hypoxia treatment level of 5 mg/l used in the current study while Pcrit for the silver-eyed stage was likely higher than the level of hypoxic exposure (Rombough, 1988b). Therefore, metabolic depression at the silver-eyed stage was likely observed as embryos attempted to balance a limited oxygen supply with a reduction in demand.

### Post-hatch **RMR**

There were persistent effects of developmental exposure to a warm temperature and hypoxia on the RMR of alevins and fry. RMR represents the energy costs necessary for self-maintenance and growth in embryos and is linked to key traits including fitness, growth and survival (Burton *et al.*, 2011). Variation in metabolic rate can also underlie differences in foraging, swimming ability and behavioural traits such as dominance, aggression and risk-behaviours (Metcalfe *et al.*, 2015). It is difficult, however, to assess whether the reduced RMR observed in numerous treatments at the alevin stage and in fry that experienced chronic developmental stressors would be beneficial or harmful to later life stages because the effects of changes in metabolic rate can be highly context dependent in terms of the habitat, food availability and temperatures present (Reid *et al.*, 2012; Auer *et al.*, 2018). Additionally, metabolic performance can vary depending on the measurement temperature. The beneficial acclimation hypothesis predicts that if developmental acclimation to a warmer temperature resulted in persistent changes in metabolic physiology, fish reared at warmer temperatures would exhibit a better performance when re-exposed to warmer temperatures, although this is not always the case (Wilson and Franklin, 2002). In this study, RMR of all post-hatch stages were tested under the same control conditions at 10°C to evaluate persistent differences across prior acclimation treatments, but the treatments reared at 14°C may have had a different metabolic performance if tested under warmer temperatures as thermal reaction norms and effects of temperature exposure depend on the testing temperature (Scott and Johnston, 2012; Pilakouta *et al.*, 2020).

### Acute stress tolerance

Carry-over effects of warm temperature exposure during early development on thermal tolerance, both persistent and latent, are not well understood in fishes (Vagner *et al.*, 2019). When CTMax was measured in fish that experienced a higher temperature during development followed by acclimation to a control temperature, CTMax decreased in sockeye salmon (Chen *et al.*, 2013) and did not change in spiny damselfish (Spinks *et al.*, 2019). In contrast, after acclimating adult zebrafish to three temperatures, fish that were reared at warmer temperatures had a consistently higher CTMax at each acclimation temperature compared to fish that were reared at lower temperatures (Schaefer and Ryan, 2006). In the current study, there were persistent effects of developmental conditions on fry thermal tolerance that were stressor and stage specific. Treatments that experienced the warmer temperature during embryogenesis had a significantly higher CTMax at the fry stage, and this was particularly evident in embryos that experienced the late warm treatment. Stage-specific responses and sensitivity to higher temperatures during development could be influenced by differences in cellular stress defence mechanisms such as the heat shock protein response, which can vary over development (Whitehouse *et al.*, 2017). Similar studies in other ectotherms, including invertebrates (Healy *et al.*, 2019) and amphibians (Mueller *et al.*, 2019) also found increases in the thermal tolerance of later life stages after exposure to warm temperatures during development. Further research on the persistent effects of developmental warm temperature exposures on juvenile fish thermal tolerance is needed to understand the variability across fish species and life stages as the carry-over effects of embryonic temperature may become more important in the thermal tolerance of species under temperatures that are predicted to increase and become more variable with climate change.

Developmental exposure to hypoxia was reported to improve tolerance to hypoxia at later stages in zebrafish (Robertson *et al.*, 2014), but in some species early exposure to hypoxia reduced hypoxia tolerance at later stages (Wood *et al.*, 2017; Cadiz *et al.*, 2018) or had no effect (Vanderplancke *et al.*, 2015; Levesque *et al.*, 2019). In this study, hypoxia tolerance at the fry stage was not affected by developmental exposure to hypoxia or to the combination of warm temperature and hypoxia; however, fry that experienced chronic warm water as embryos had a significantly higher hypoxia tolerance. Similarly, common sole larvae exposed to a warm temperature were more tolerant to hypoxia as juveniles (Zambonino-Infante *et al.*, 2013). Oxygen supply and demand underlie physiological responses to hypoxia. Similar to what was observed in killifish (McBryan *et al.*, 2016), the reduced RMR of the chronic warm treatment at the fry stage may have improved hypoxia tolerance by reducing oxygen demand and therefore making the fry more tolerant of drops in oxygen tension.

### Future directions and conclusions

It remains unclear how long the effects of early exposure to hypoxia or warming can persist through later life stages of fishes. For example, early developmental exposure to hypoxia did not confer lasting effects on hypoxia tolerance, acclimation capacity or aerobic metabolic performance in juvenile Atlantic salmon reared in hypoxia when tested after 8–15 months in normoxia (Wood *et al.*, 2017; Wood *et al.*, 2020). However, even if the persistent effects observed in this study do not carry-over to later juvenile stages, the persistent effects we observed in performance at the fry stage can still impact individual survival in the river. Effects at the fry stage can be important as Central Valley juvenile Chinook salmon commonly leave their natal tributaries and begin their outmigration as fry (Williams, 2001, 2006), and even small differences in fry survival rates can impact adult recruitment (Sturrock *et al.*, 2020).

In conclusion, developmental exposure to warm water and hypoxia as individual or combined stressors can affect the development and physiology of Chinook salmon both during embryonic exposure and in later life stages following hatch from the gravel redds. Lethal thermal thresholds for salmon embryos are currently the focus of many management strategies (Danner *et al.*, 2012); however, the persistent effects of developmental warm temperature exposures on acute stress tolerance and metabolic performance suggest that sublethal effects experienced early in development have lasting effects and therefore are important management considerations, particularly as climate change progresses. The differences between the multiple stressor and single stressor treatments in hatching success, RMR, thermal tolerance and developmental rate highlight the value of investigating multiple stressor interactions that cannot be inferred from single stressors alone. Additionally, the effects of hypoxia with and without warming provide further evidence that DO levels and their interaction with temperature affect Chinook salmon survival, physiology and growth, providing evidence that monitoring hypoxia should be integrated into management practices. While we were unable to test all stages of embryonic development, and therefore cannot conclusively identify a critical developmental window, our results show that the effects of environmental stressors on salmon development are both stage and stressor dependent. Further research is needed across more developmental stages and different levels of each stressor to identify critical windows of stressor sensitivity in salmon development. Additionally, the short- and long-term effects of a warmer temperature and hypoxia on salmon embryo development should be investigated in field settings to test whether developmental plasticity and carry-over effects in environmentally complex field settings are consistent with those observed in laboratory studies. As we incorporate more environmental complexity and life history transitions into our experimental designs it is important to accurately capture these complexities in our empirical work. Depending on the watershed, sources of variability within redds (e.g. ground water intrusion) as well as in the water column above (e.g. due to changes in river flows) are important considerations for understanding when critical windows of vulnerability match periods of poor water quality across seasons. An understanding of where managers have the capacity to mitigate water quality will be important for applying what is learned from these types of studies in conservation physiology to management solutions.

## Funding

This work was funded by the US Bureau of Reclamation in partnership with the Delta Science Council Delta Science Program and California Sea Grant (grant number R16AC00026 awarded to A.M.D.) and the California Agricultural Experimental Station of the University of California Davis (grant numbers CA-D-ASC-2252-H to A.E.T. and CA-D-ASC-2098 to N.A.F.).

## Supplementary Material

Del_Rio_et_al_Supplementary_Material_060921_Final_coab054
