# Supplementary material for: Differential sensitivity to warming and hypoxia during development and long-term effects of developmental exposure in early life stage Chinook salmon
Source: Conserv Physiol. 2021 Jul 8;9(1):coab054. doi: 10.1093/conphys/coab054 (PMC8271147; doi:10.1093/conphys/coab054)
Supplement: Del_Rio_et_al_Supplementary_Material_060921_Final_coab054 [file del_rio_et_al_supplementary_material_060921_final_coab054.docx]

**Supplementary material to “Differential sensitivity to warming and hypoxia during development and long-term effects of developmental exposure in early life stage Chinook salmon”**

**Table S1.** Water temperature (°C) and dissolved oxygen (DO, mg/l and % saturation) in each treatment for both control conditions and during chronic or short term stressor exposures.

Water temperature was measured daily in each culture bucket and is reported as the average between the replicates for each treatment (±SD). Dissolved oxygen was measured daily in each culture bucket and is reported as the average of the three replicates

per treatment (±SD).

|  | **Control conditions** | | | **Exposure conditions** | | |
| --- | --- | --- | --- | --- | --- | --- |
| **Treatment** | **Temperature °C** | **DO (mg/l)** | **DO (% saturation)** | **Temperature °C** | **DO (mg/L)** | **DO (% saturation)** |
| Control | 10.0 ± 0.4 | 10.8 ± 0.3 | 96.9 ± 1.5 |  |  |  |
| Early hypoxia | 10.0 ± 0.6 | 10.7 ± 0.7 | 96.4 ± 6.5 | 10.2 ± 0.6 | 5.8 ± 1.02 | 52 ± 9.8 |
| Late hypoxia | 10.0 ± 0.4 | 10.8 ± 0.3 | 96.2 ± 2.1 | 10.0 ± 0.2 | 5.6 ± 0.8 | 49.4 ± 7.2 |
| Chronic hypoxia | 10.2 ± 0.5 | 10.7 ± 0.4 | 96.4 ± 3.4 | 10.1 ± 0.3 | 5.9 ± 0.9 | 52.8 ± 8.1 |
| Early warm | 10.1 ±0.3 | 10.77 ± 0.2 | 96.5 ± 1.5 | 13.9 ± 0.5 | 9.7 ± .02 | 94.8 ± 1.4 |
| Late warm | 10.0 ± 0.3 | 10.8 ± 0.7 | 96.7 ± 1.4 | 14.1 ± 0.6 | 10.0 ± 0.2 | 95.9 ± 0.9 |
| Chronic warm | 10.0 ± 0.4 | 10.8 ± 0.3 | 96.3 ± 2.0 | 14.1 ± 0.6 | 9.8 ±0.2 | 95.1 ± 1.5 |
| Early warm hypoxia | 10.2 ± 0.5 | 10.8 ± 0.4 | 96.7 ± 2.8 | 14.1 ± 0.5 | 5.5 ± 1.0 | 55.3 ± 7.2 |
| Late warm hypoxia | 10.1 ± 0.7 | 10.7 ± 0.9 | 95.5 ± 7.12 | 14.2 ± 0.4 | 5.8 ± 0.9 | 55.8 ± 6.5 |
| Chronic warm hypoxia | 9.9 ± 0.4 | 10.8 ± 0.3 | 96.4 ± 2.4 | 14.2 ± 0.6 | 5.2 ± 0.6 | 51.6 ± 5.7 |

**
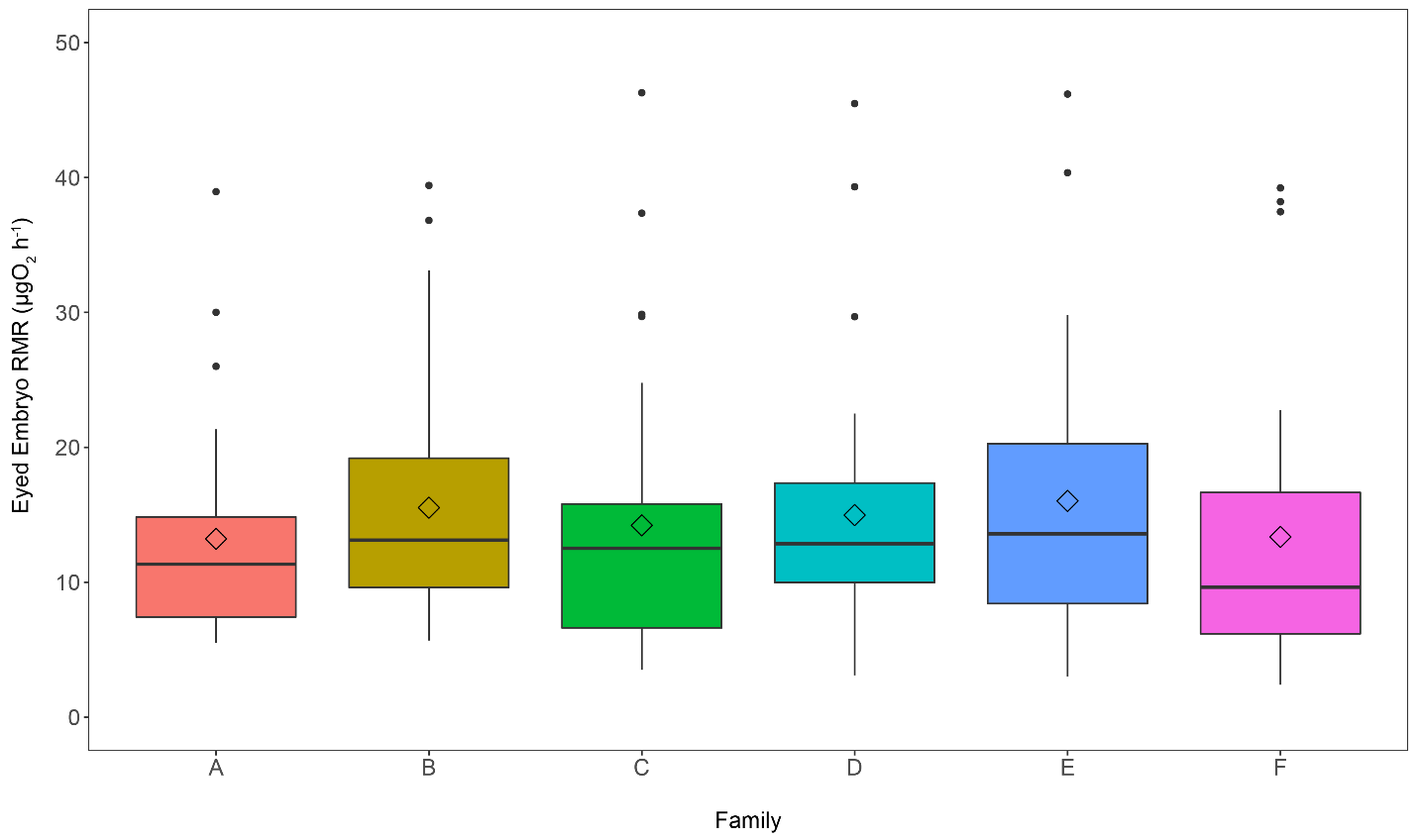
**

**Figure S1.** Routine metabolic rate in µgO_2_ h^-1^ per embryo at the eyed stage grouped by family across treatments. Boxplots of the data represent the median as the center line, interquartile range (IQR) as the box, values 1.5 times the IQR as the whiskers, and values greater than 1.5 times the IQR as black points. Diamonds indicate the mean value. Colors represent the family.

**
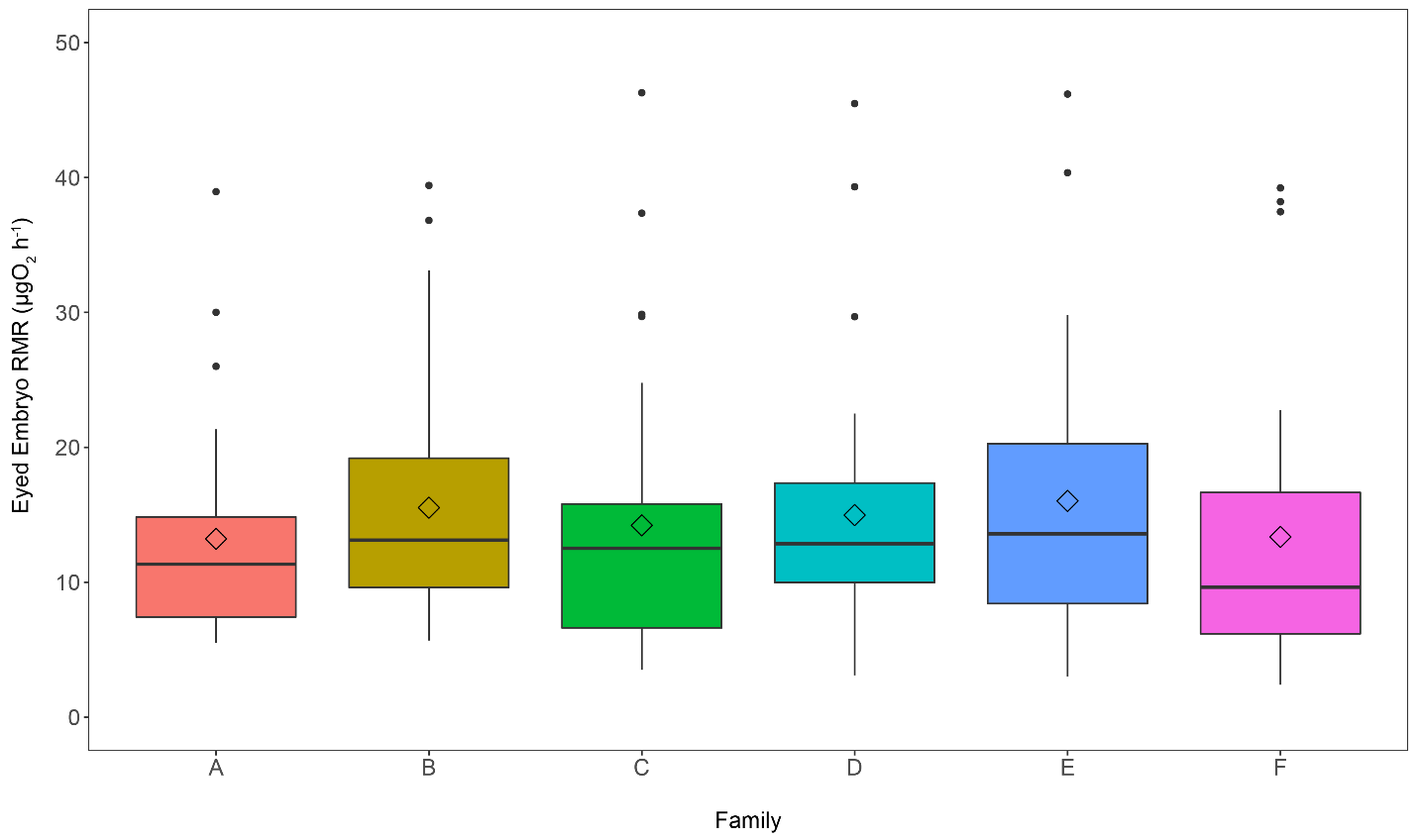
**

**Figure S2.** Routine metabolic rate in µgO_2_ h^-1^ per embryo at the silver eyed stage grouped by family across treatments. Boxplots of the data represent the median as the center line, interquartile range (IQR) as the box, values 1.5 times the IQR as the whiskers, and values greater than 1.5 times the IQR as black points. Diamonds indicate the mean value. Colors represent the family.

**
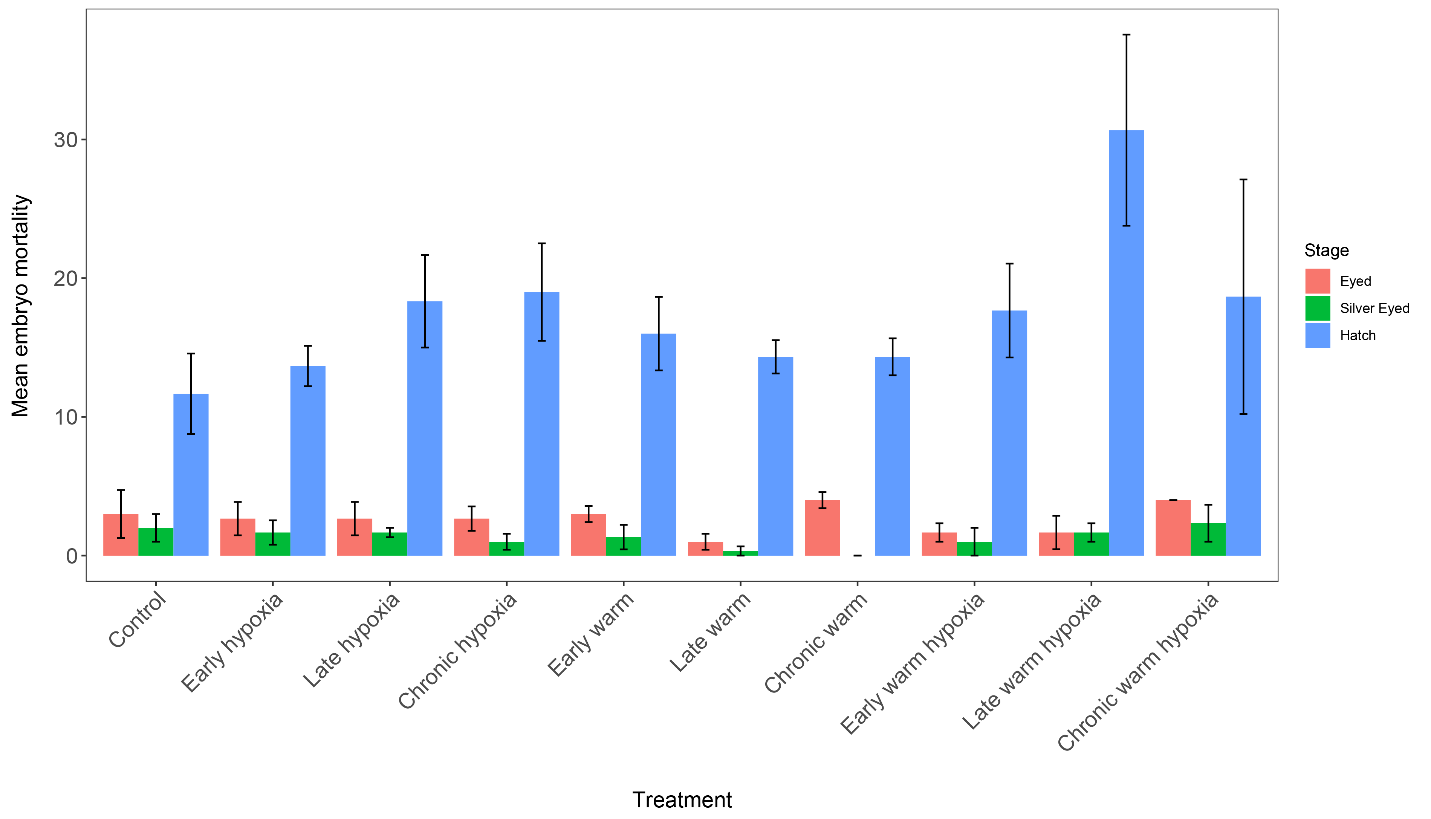
**

**Figure S3.** Mean embryo mortality between each developmental stage (n=3 replicates per treatment). Mortality was counted as the number of embryos that died between fertilization and the eyed stage, eyed stage to silver eyed stage, and silver eyed stage through hatching. Note that the developmental time periods were different lengths and the time between the eyed and silver eyed stage was often the shortest. The total number of embryos also decreased as development progressed as 18 embryos were removed from each treatment for respirometry at both the eyed and silver eyed stages.


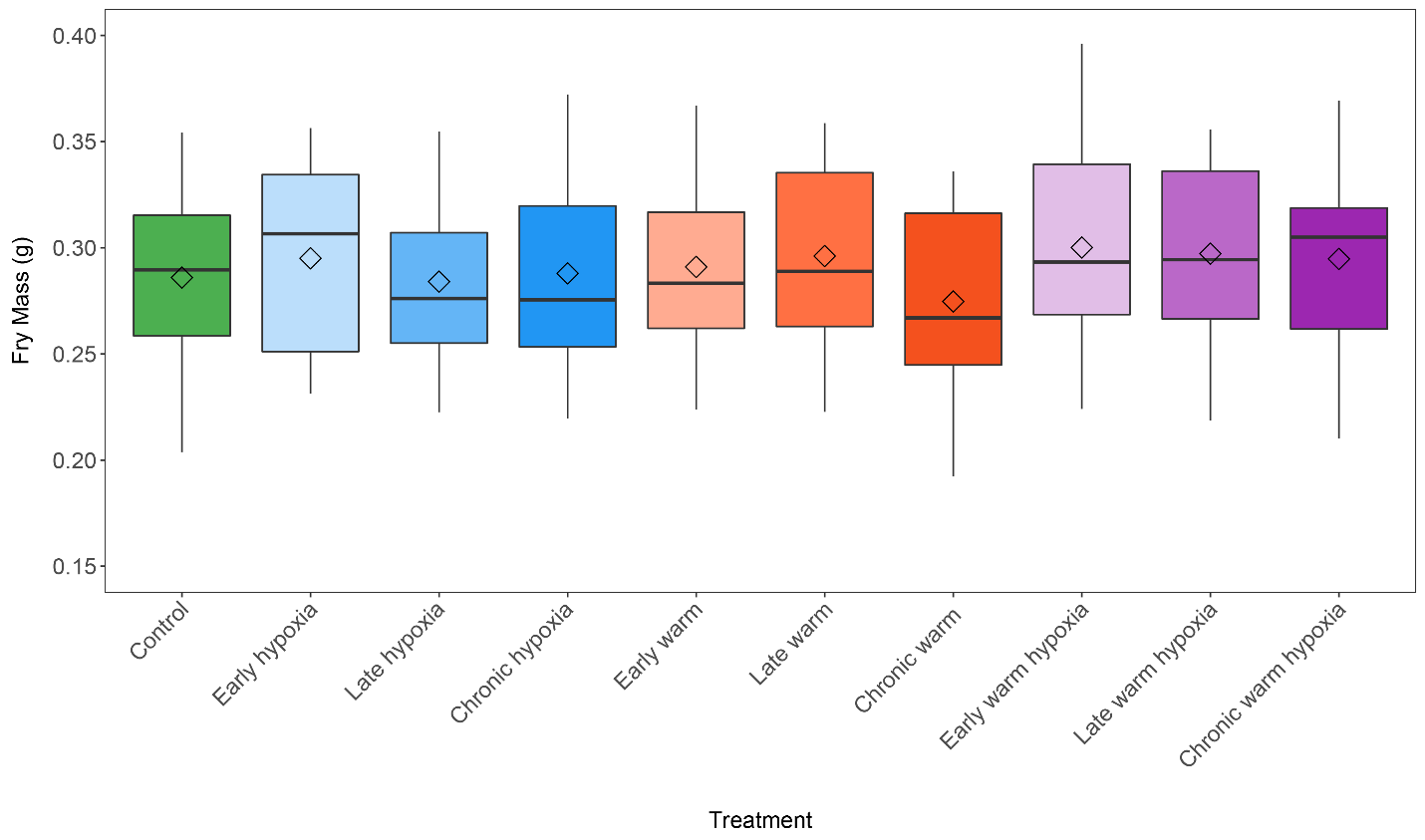


**Figure S4**. Mass (g) at the fry stage represented by boxplots (n= 47 per treatment except late hypoxia and late warm hypoxia [n=45], early hypoxia and chronic warming [n=46], and early warming and early warm hypoxia [n=48]). Boxplots include the median as the center line, interquartile range (IQR) as the box, values 1.5 times the IQR as the whiskers, and values greater than 1.5 times the IQR as black points. Diamonds indicate the mean values. Colors represent the developmental treatment: control (green, 100% DO, 10°C), hypoxia (blue, 50% DO, 10°C), warming (red, 100% DO, 14°C), or warming and hypoxia (purple, 50% DO, 14°C). The three boxplots for each stressor type represent the developmental timing of exposure as early (eyed stage, light shade color), late (silver eyed stage, medium shade color) and chronic (fertilization through hatching, dark color shade).


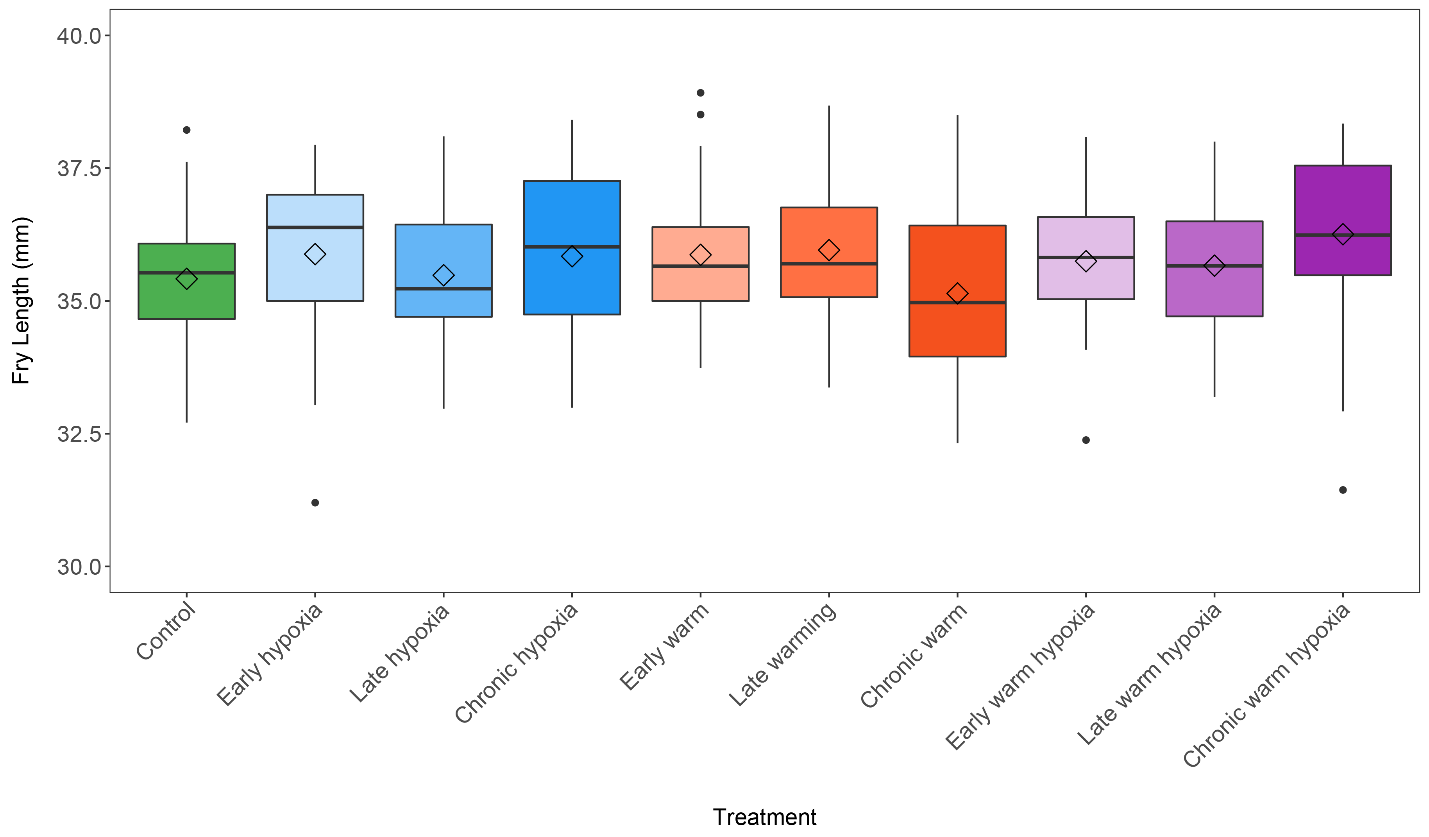


**Figure S5**. Length (mm) at the fry stage represented by boxplots (n= 47 per treatment except late hypoxia and late warm hypoxia [n=45], early hypoxia and chronic warming [n=46], and early warming and early warm hypoxia [n=48]). Boxplots include the median as the center line, interquartile range (IQR) as the box, values 1.5 times the IQR as the whiskers, and values greater than 1.5 times the IQR as black points. Diamonds indicate the mean values. Colors represent the developmental treatment: control (green, 100% DO, 10°C), hypoxia (blue, 50% DO, 10°C), warming (red, 100% DO, 14°C), or warming and hypoxia (purple, 50% DO, 14°C). The three boxplots for each stressor type represent the developmental timing of exposure as early (eyed stage, light shade color), late (silver eyed stage, medium shade color) and chronic (fertilization through hatching, dark color shade).
